# Evaluation of *In Vitro* Activity of the Class I PI3K Inhibitor Buparlisib (BKM120) in Pediatric Bone and Soft Tissue Sarcomas

**DOI:** 10.1371/journal.pone.0133610

**Published:** 2015-09-24

**Authors:** Jennifer L. Anderson, Ann Park, Ryan Akiyama, William D. Tap, Christopher T. Denny, Noah Federman

**Affiliations:** 1 Department of Pediatrics, Division of Hematology/Oncology, Gwynne Hazen Cherry Memorial Laboratories, University of California, Los Angeles, Los Angeles, California, United States of America; 2 Department of Medicine, Division of Solid Tumors, Sarcoma Medical Oncology Service, Memorial Sloan Kettering Cancer Center, New York, New York, United States of America; 3 Department of Medicine, Weill Cornell Medical College, New York, New York, United States of America; 4 Molecular Biology Institute, University of California, Los Angeles, Los Angeles, California, United States of America; 5 Jonsson Comprehensive Cancer Center, University of California, Los Angeles, Los Angeles, California, United States of America; 6 California NanoSystems Institute, University of California, Los Angeles, Los Angeles, California, United States of America; Johns Hopkins University, UNITED STATES

## Abstract

Pediatric bone and soft tissue sarcomas often display increased Akt phosphorylation through up regulation of insulin-like growth factor (IGF1) signaling. Additionally, Akt signaling has been linked to resistance to IGF1 receptor (IGF1R) and mTOR (mammalian target of rapamycin) inhibitors in sarcoma, further demonstrating the role of Akt in tumor survival. This suggests targeting components of the PI3K/Akt pathway may be an effective therapeutic strategy. Here, we investigated the *in vitro* activity of the pan-class I PI3K inhibitor buparlisib (BKM120) in pediatric bone and soft tissue sarcomas. Buparlisib inhibited activation of Akt and signaling molecules downstream of mTORC1 (mTOR complex 1) in Ewing sarcoma, osteosarcoma, and rhabdomyosarcoma cell lines. Anti-proliferative effects were observed in both anchorage dependent and independent conditions and apoptosis was induced within 24 hours of drug treatment. Buparlisib demonstrated cytotoxicity as a single agent, but was found to be more effective when used in combination. Synergy was observed when buparlisib was combined with the IGF1R inhibitor NVP-AEW541 and the mTORC1 inhibitor rapamycin. The addition of NVP-AEW541 also further reduced phospho-Akt levels and more potently induced apoptosis compared to buparlisib treatment alone. Additionally, the combination of buparlisib with the MEK1/2 inhibitor trametinib resulted in synergy in sarcoma cell lines possessing MAPK pathway mutations. Taken together, these data indicate buparlisib could be a novel therapy for the treatment of pediatric bone and soft tissue sarcomas.

## Introduction

Pediatric bone and soft tissue sarcomas have an overall 5-year survival rate near 60% that has plateaued with current treatments. Patients with recurrent and metastatic disease have a worse prognosis, indicating the need for novel therapeutic strategies. Advancements in the understanding of the molecular pathogenesis of these malignancies have led to the to the evaluation of molecularly targeted therapies.

While pediatric sarcomas have diverse underlying mutations, they often converge on common signaling pathways that regulate cellular growth and survival. For example, both Ewing sarcoma and alveolar rhabdomyosarcoma display a dependence on insulin-like growth factor (IGF) signaling despite harboring distinct oncogenic fusions [1]. Promising phase I data, particularly in Ewing sarcoma, led to the phase II evaluation of IGF1 receptor (IGF1R) inhibitors in multiple sarcoma subtypes. However, phase II responses were only observed in a subset of patients and drug resistance often occurred [2–6]. In rhabdomyosarcoma xenograft models, tumors that escaped IGF1R inhibition displayed reactivation of Akt despite sustained down regulation of IGF1R activity [7]. Increased Akt phosphorylation was also observed in anti-IGF1R resistant Ewing sarcoma cell lines [8] and tumors [9]. Additionally, mTOR (mammalian target of rapamycin) inhibition induces activation of Akt in cancer cell lines and patient tumors [10, 11]. This indicates a dependence on Akt signaling for cell survival as well as escape from targeted therapies.

Akt is activated upon phosphoinositide 3-kinase (PI3K)-mediated modulation of phospholipids within the plasma membrane. Three classes of PI3Ks are activated by various stimuli and modify distinct lipid substrates. Class IA PI3Ks are heterodimeric proteins consisting of a regulatory (p85) and catalytic (p110) subunit with several isoforms. The three catalytic isoforms (p110α, p110β, p110δ) possess differential signaling functions, but with some degree of redundancy [12]. In general, class IA PI3Ks act downstream of growth factor receptor tyrosine kinases to phosphorylate phosphatidylinositol-4,5-bisphosphate (PIP_2_) and generate phosphatidylinositol-3,4,5-trisphosphate (PIP_3_) at the cell membrane to create a docking site for signaling proteins. The phosphatase PTEN (phosphatase and tensin homolog) antagonizes PI3K signaling by dephosphorylating PIP_3_ to PIP_2_. Accumulation of PIP_3_ recruits Akt to the cell membrane, where it is first phosphorylated within the activation loop at threonine 308 by phosphoinositide-dependent protein kinase 1 (PDK1). Akt is then phosphorylated within a hydrophobic motif at serine 473 by mTOR complex 2 (mTORC2), which results in maximum kinase activity [13]. Akt-mediated activation of mTOR complex 1 (mTORC1) then causes increased cellular proliferation by phosphorylating proteins such as p70 S6 kinase (S6K) and 4E-binding protein 1 (4E-BP1) that regulate protein synthesis [12]. In cancer, this pathway can be constitutively activated, most commonly by loss of PTEN or an activating mutation in the *PIK3CA* gene that encodes the p110α catalytic subunit of PI3K.

Several agents that target the PI3K/Akt pathway are currently in clinical development. These agents include mTOR inhibitors (rapamycin analogs and active site mTOR inhibitors), PI3K inhibitors (pan-class I and isoform specific), dual PI3K/mTOR inhibitors, and Akt inhibitors [14]. Early phase I studies of the pan-class I PI3K inhibitor buparlisib demonstrated a clinical benefit in breast cancer [15, 16], leading to a multi-center phase III trial to evaluate progression free survival in patients stratified by PI3K pathway activating mutations [17]. More dramatic responses have been observed with PI3K inhibition in B-cell malignancies. The p110δ PI3K inhibitor idelalisib displayed an overall response rate of 72% as a single agent [18] and 81% when combined with rituximab [19] in chronic lymphocytic leukemia. Additionally, pre-clinical evaluation of drug combinations revealed synergy between Bruton’s tyrosine kinase (BTK) and PI3K inhibition in diffuse large B-cell lymphoma [20]. While various rapamycin analogs have been clinically evaluated in sarcoma, less work has been done to explore the efficacy of agents directed against other molecules in the PI3K/Akt pathway.

Buparlisib (BKM120) selectively targets the catalytic isoforms of class IA (p110α, p110β, p110δ) and class IB (p110γ) PI3Ks [21]. It is at least 50-fold more specific to PI3K than other protein kinases and potently blocks activation of Akt and other downstream signaling proteins. The ability of buparlisib to inhibit proliferation and induce apoptosis in tumor cell lines has led to clinical testing for various solid tumors and hematological malignancies. In this study, we evaluated the *in vitro* activity of the pan-class I PI3K inhibitor buparlisib alone and in combination with other targeted agents in three common pediatric sarcomas: Ewing sarcoma (ES), osteosarcoma (OS), and rhabdomyosarcoma (RMS).

## Methods

### Cell culture

ES cell lines (TC-174, TC-32, A4573, 6647, A673, 5838, RDES, SKES, SK-N-MC, TC-248, TTC-475), MCF-7 cells, and LNCaP cells were cultured in Iscove’s modified Dulbecco’s medium (IMDM) containing 10% fetal bovine serum (FBS). OS (KHOS, KHOS 240S, HOS, MNNG, KHOS 312H, OS 187, SJSA, HT161) and RMS cell lines (RD, A204, SJCRH30) were cultured in Dulbecco’s Modified Eagle Medium (DMEM) containing 10% FBS. Cell lines were either purchased from ATCC or were a gift from Timothy J. Triche, MD, PhD at the Saban Research Institute, Children’s Hospital Los Angeles. Cell line authentication including PI3K mutational analysis was performed as previously described [22]. PI3K mutational status for sarcoma cell lines was also obtained from the Catalogue Of Somatic Mutations In Cancer (http://cancer.sanger.ac.uk) [23].

### Reagents

Buparlisib (BKM120) was provided by MTA from Novartis Pharma (Basel, Switzerland) or obtained from Selleck Chemical. Doxorubicin HCl was obtained from Shandong Tianyu Fine Chemical Co., Ltd. NVP-AEW541 was obtained from Cayman Chemical. Rapamycin was obtained from Cell Signaling Technology. Trametinib and pictilisib (GDC-0941) were obtained from Selleck Chemical.

### Immunoblot

Cells were incubated for approximately one hour on ice in lysis buffer (50 mM Tris pH 7.6, 0.5% NP-40, 10% glycerol, 30 mM NaCl, 1 mM EDTA) supplemented with Complete Mini EDTA-free protease inhibitor cocktail (Roche), 1 mM Na_3_VO_4_, and 1 mM NaF. Lysates were combined with 6X protein sample buffer (0.35 M Tris pH 6.8, 10% SDS, 30% glycerol, 0.6 M DTT, 0.012% bromophenol blue) and boiled for 5–10 minutes prior to loading on an 8–10% polyacrylamide gel. The primary antibodies used for these studies were rabbit anti-PTEN, rabbit anti-phospho-Akt (Ser473), rabbit anti-phospho-Akt (Thr308), mouse anti-Akt (pan), rabbit anti-phospho-p44/42 MAPK (Erk1/2) (T202/T204 on Erk1, T185/T187 on Erk2), mouse anti-p44/42 MAPK (Erk1/2), mouse anti-phospho-p70 S6 Kinase (Thr389), rabbit anti-p70 S6 Kinase, rabbit anti-phospho-4E-BP1 (Thr37/46), rabbit anti-4E-BP1, rabbit anti-STAT3 (Y705), mouse anti-STAT3, and rabbit anti-cleaved PARP from Cell Signaling Technology; and mouse anti-β-actin from Sigma. Secondary antibodies conjugated to HRP were sheep anti-mouse IgG from GE Healthcare and goat anti-rabbit IgG from Santa Cruz Biotechnology. Secondary antibodies conjugated to infrared dyes were IRDye 800CW goat anti-mouse IgG and IRDye 680RD goat anti-rabbit IgG from LI-COR Biosciences. Fluorescent westerns were imaged using the Odyssey Infrared Imaging System (LI-COR Biosciences). Signals were quantified by measuring the integrated intensity values of each band using Odyssey software (LI-COR Biosciences).

### Cell viability assay

Cells were seeded in 96-well plates and drugs were added after overnight incubation, with each treatment condition performed in triplicate. After drug treatment, 10 μl of 5 mg/ml MTT (3-(4,5-dimethylthiazolyl-2)-2, 5-diphenyltetrazolium bromide) in PBS was added to cells and allowed to incubate for 2–4 hours at 37°C. Cells were then lysed with 100 μl of 15% SDS in 15 mM HCl and incubated overnight at room temperature in the dark. Plate absorbance was read at 595 nm using a Bio-rad microplate reader. Percent viability was calculated by normalizing absorbance values to those from cells grown in media without drug after background subtraction. IC50 values were calculated by fitting dose-response curves to a four-parameter, variable slope sigmoid dose-response model (Prism Software, GraphPad). Synergistic, additive, or antagonistic effects of buparlisib combination treatment were determined based on combination indices and isobologram plots generated with CompuSyn software (ComboSyn, Inc., Paramus, NJ) using the method of Chou and Talalay [24].

### Soft agar assay

Anchorage independent growth was measured by seeding 1000–10,000 cells in 6 well plates or 500 cells in 12 well plates in 0.3% agar supplemented with 20% FBS with a 0.75% agar underlay. After 14 days of growth, colonies were stained with 0.005% crystal violet, imaged, and quantified using ImageJ.

### Cell cycle analysis

Cells were treated with buparlisib for 24 hours, then media containing detached mitotic, apoptotic and dead cells was pooled with trypsinized adherent cells. Cells were washed with PBS and fixed with 70% ice-cold ethanol. After incubation at -20°C overnight, cells were resuspended in propidium iodide staining solution (0.1 mg/ml propidium iodide (Sigma), 3.9 mM sodium citrate, 0.3% Triton X-100, 0.1 mg/ml ribonuclease A), incubated for 30 minutes, then analyzed using Becton Dickson (BD) FACScan or LSR II flow cytometry analyzers. 10,000 live cell events were recorded after gating cells using FL2-W and FL2-A to discriminate between G1 doublet and G2/M single cell populations. Percentage of cells in G0/G1, S, and G2/M phases of the cell cycle was calculated using ModFit LT 4.1 (Verity Software).

## Results

### Buparlisib inhibits the activity of signaling molecules downstream of PI3K

**Fig 1:**
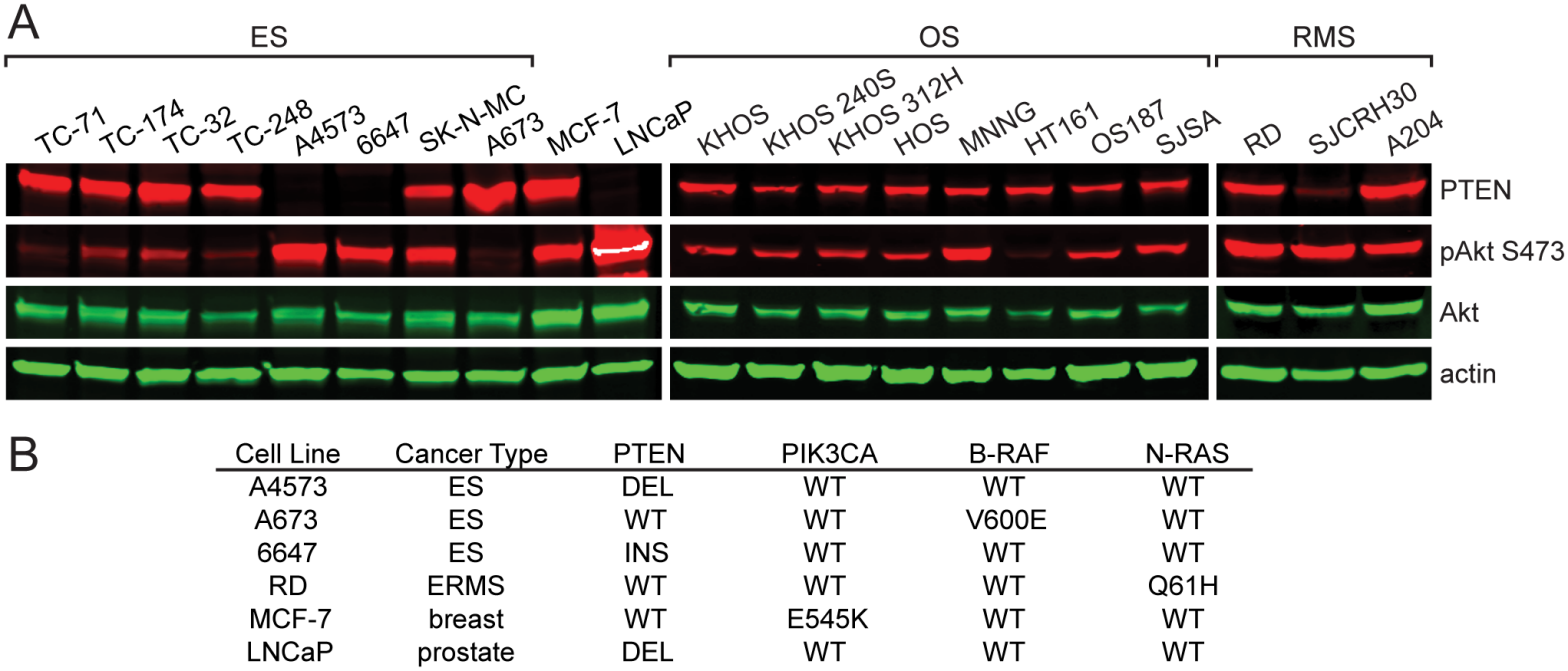
PTEN and basal phospho-Akt levels in a panel of tumor cell lines. (**A**) Immunoblot analysis of PTEN, phospho-Akt (S473), total Akt, and actin in ES, OS, RMS, breast cancer (MCF-7), and prostate cancer (LNCaP) cell lines. (**B**) Known PI3K and MAPK pathway mutations in cell lines evaluated in A. ES, Ewing sarcoma; ERMS, embryonal rhabdomyosarcoma; DEL, deletion; INS, insertion.

We first assessed PTEN expression and basal levels of Akt phosphorylation in a panel of ES, OS, and RMS cell lines. The breast cancer cell line MCF-7 and prostate cancer cell line LNCaP were also included since they display increased Akt phosphorylation due the presence of *PIK3CA* and *PTEN* mutations, respectively. Akt phosphorylation was detected at varying levels in the majority of sarcoma cell lines examined, with higher levels observed in ES cell lines that lacked PTEN expression (Fig 1A). The ES cell line A673, which harbors a B-RAF mutation and thus expected to have less dependence on PI3K/Akt signaling, displayed low phospho-Akt levels. Conversely, the RMS cell line RD displayed high levels of Akt phosphorylation despite possessing a MAPK pathway mutation (Fig 1A and 1B). This is likely the result of the ability of the p110α isoform of PI3K to function as downstream effector of oncogenic RAS [25].

**Fig 2:**
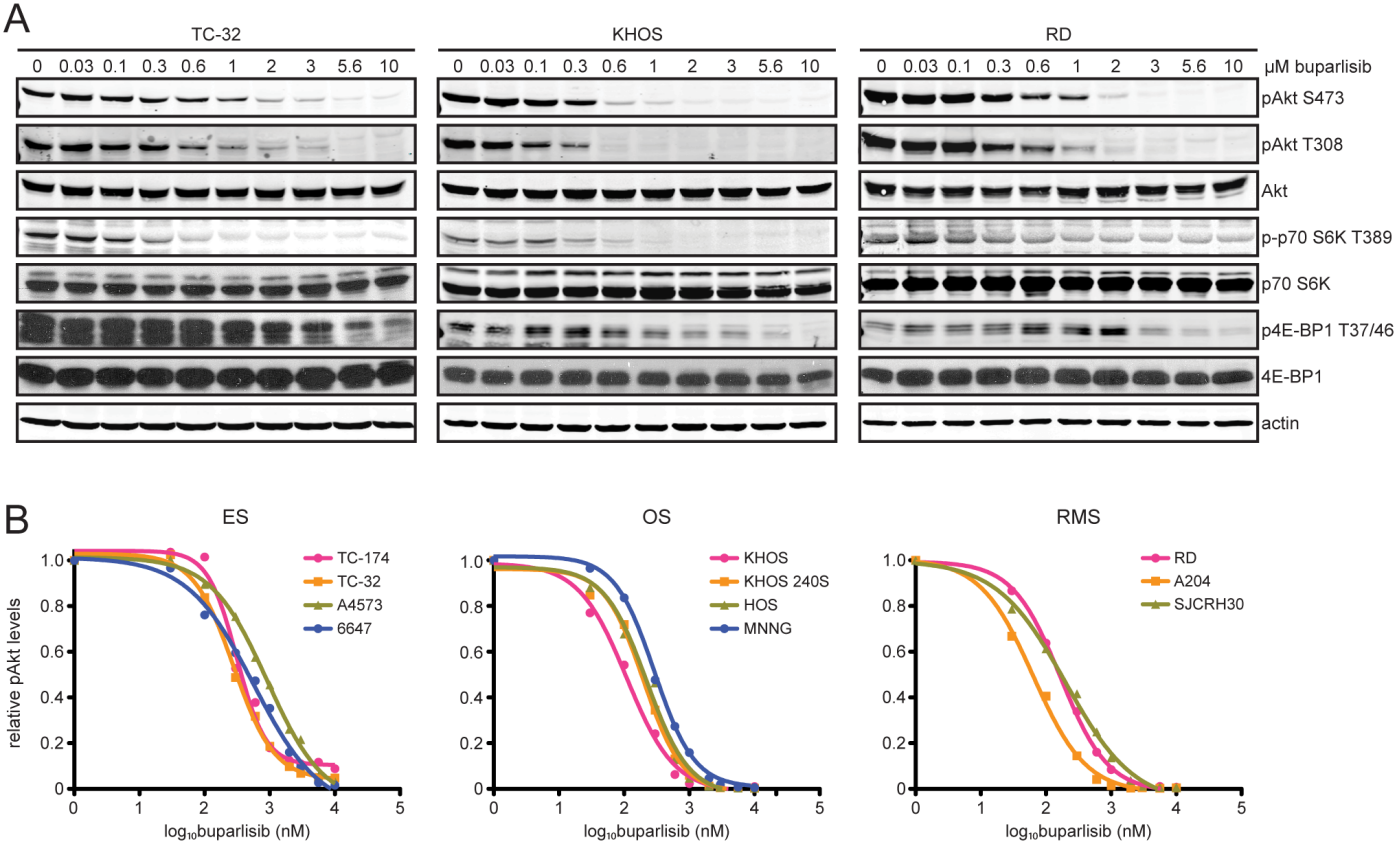
Buparlisib treatment reduces phosphorylation of signaling molecules downstream of PI3K and mTORC1. (**A**) Immunoblot analysis of phospho-Akt (S473 and T308), total Akt, phospho-p70 S6K (T389), total p70 S6K, phospho-4E-BP1 (T37/46), total 4E-BP1, and actin in ES (TC-32), OS (KHOS), and RMS (RD) cells treated with increasing concentrations of buparlisib for one hour. (**B**) Phospho-Akt (S473) and total Akt levels were quantitated based on Odyssey software integrated intensity values. Phospho-Akt levels were normalized to total Akt levels, then normalized to cells treated with DMSO in order to determine relative phospho-Akt levels. Relative phospho-Akt levels were plotted against log buparlisib concentration and IC50 values were calculated by fitting this data to a four-parameter, variable slope sigmoid dose-response model. Each data point represents the average of at least three independent experiments.

We next evaluated the ability of buparlisib to inhibit cellular signaling downstream of PI3K. Increasing doses were added to cells and phosphorylation of signaling molecules was examined by immunoblot after one hour of treatment (Fig 2A). Phosphorylation of Akt was inhibited at both serine 473 and threonine 308. Quantitative immunoblot analysis was used to measure levels of Akt phosphorylation at serine 473 (Fig 2A). Phospho-Akt levels were normalized to levels of total Akt and fit to dose-response curves to calculate the half maximal inhibitory concentration (IC50) (Fig 2B). IC50 values for ranged from 64 to 916 nM, with the largest values occurring in cell lines that lack PTEN expression (S1 Table). Buparlisib also inhibited the phosphorylation of proteins downstream from mTORC1, such as S6K and 4E-BP1 (Fig 2A). Phosphorylation of S6K was more potently inhibited by buparlisib. Inhibition of phospho-4E-BP1 was only observed at high buparlisib concentrations.

**Fig 3:**
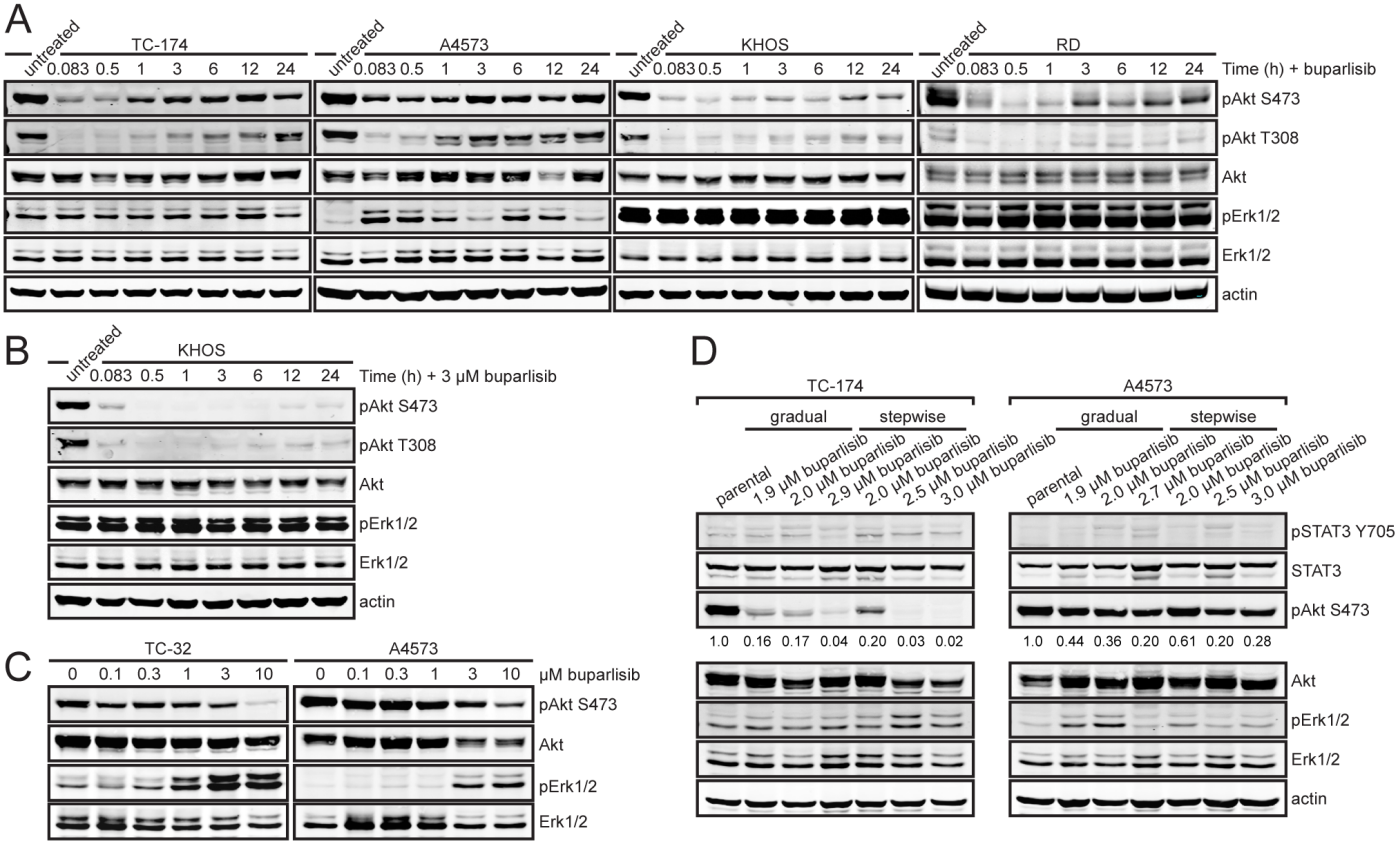
Re-activation of Akt and induction of Erk occur within 24 hours of buparlisib treatment. (**A**) Immunoblot analysis of phospho-Akt (S473), total Akt, phospho-Erk1/2 (T202/T204 on Erk1, T185/T187 on Erk2), total Erk, and actin in ES (TC-174, A4573), OS (KHOS), and RMS (RD) cells after treatment with buparlisib for periods of 5 minutes to 24 hours. TC-174, KHOS, and RD cells were treated with 1 μM buparlisib. A4573 cells were treated with 3 μM buparlisib. (**B**) Immunoblot analysis of phospho-Akt (S473), total Akt, phospho-Erk1/2 (T202/T204 on Erk1, T185/T187 on Erk2), total Erk, and actin in KHOS cells after treatment with 3 μM buparlisib for periods of 5 minutes to 24 hours. (**C**) Immunoblot analysis of phospho-Akt (S473), total Akt, phospho-Erk1/2 (T202/T204 on Erk1, T185/T187 on Erk2), and total Erk in TC-32 and A4573 cells with increasing concentrations of buparlisib for 24 hours. (**D**) Immunoblot analysis of phospho-STAT3 (Y705), total STAT3, phospho-Akt (S473), total Akt, phospho-Erk1/2 (T202/T204 on Erk1, T185/T187 on Erk2), total Erk, and actin in TC-174 and A4573 parental cells and cells passaged in increasing concentrations of buparlisib. Phospho-Akt levels were quantitated based on Odyssey software integrated intensity values. Values are listed below each band.

We also performed a time course of buparlisib treatment to examine the dynamics of Akt inhibition. Cells were treated with buparlisib for periods of five minutes up to 24 hours and immunoblot analysis was utilized to measure levels of Akt and Erk phosphorylation. Akt was inhibited quickly and displayed maximum inhibition after approximately one hour of buparlisib treatment. After three hours, phospho-Akt levels increased despite continued treatment with buparlisib (Fig 3A). This pattern of Akt reactivation was also observed MCF-7 cells (S1A Fig). Increasing the concentration of buparlisib lengthened the duration of time prior to Akt reactivation and reduced the amount of phosphorylation (Fig 3B). Akt phosphorylation increased at threonine 308 and serine 473 (Fig 3A), indicating a reactivation mechanism that is upstream of both PDK1 and mTORC2. Additionally, we did not observe an increase in phospho-S6K levels over time after buparlisib treatment (S2 Fig). In some cell lines, the increase in Akt phosphorylation corresponded with an increase in phospho-Erk levels (Fig 3A). Additionally, elevated Erk phosphorylation was observed at higher concentrations of buparlisib in TC-32 and A4573 cells after 24 hours of treatment (Fig 3C).

To determine the effects of prolonged buparlisib treatment on cellular signaling, we generated drug resistant cell lines. TC-174 and A4573 cells were passaged in increasing concentrations of buparlisib. We utilized two different dose escalation schemes: a gradual dose escalation in which buparlisib concentration was increased by 100 nM increments and a stepwise dose escalation in which buparlisib concentration was doubled until reaching 1.5 μM, then increased by 500 nM increments. After approximately six months of escalating drug treatment, we generated cell lines that continued to proliferate in buparlisib concentrations up to 3.0 μM. Buparlisib resistant TC-174 cells displayed sustained down regulation of Akt phosphorylation at doses above 2.0 μM. Buparlisib resistant A4573 cells displayed reduced phospho-Akt levels compared to parental cells, but still maintained moderate levels of Akt phosphorylation. Additionally, we did not observe a marked increase in Erk or STAT3 phosphorylation after prolonged buparlisib treatment (Fig 3D).

### Buparlisib inhibits anchorage dependent and independent growth of sarcoma cell lines

**Fig 4:**
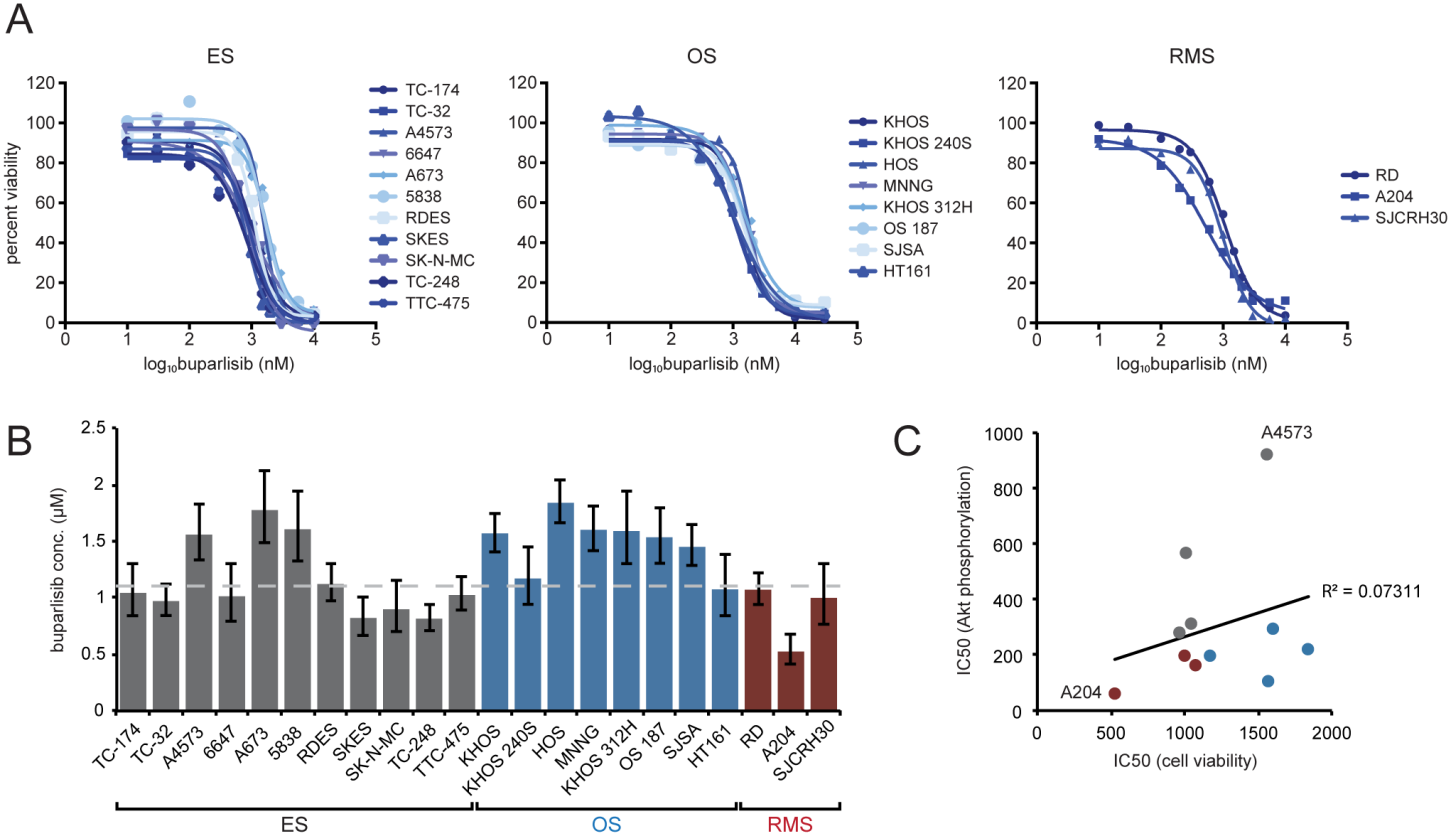
Buparlisib inhibits proliferation of pediatric sarcoma cell lines. (**A**) Cells were treated with media containing 0.1% DMSO or concentrations of buparlisib ranging from 10 nM to 10 μM for 72 hours. Cell viability was determined by MTT assay. Percent cell viability was plotted against log buparlisib concentration and IC50 values were calculated by fitting this data to a four-parameter, variable slope sigmoid dose-response model. Each point is the average of at least three independent experiments. (**B**) Cell viability IC50 values for pediatric sarcoma cell lines. Columns represent the average of at least three independent experiments, error bars represent 95% confidence intervals. Dashed line indicates median IC50. (**C**) Scatter plot showing the low correlation (R^2^ = 0.07311) between cell viability and phospho-Akt IC50 values. The cell lines that possess the lowest (A204) and highest (A4573) phospho-Akt IC50s are indicated on the graph. Dot colors indicate sarcoma type: gray—ES, blue—OS, burgundy—RMS.

After demonstrating that buparlisib inhibits PI3K/Akt signaling, we examined its anti-proliferative effects in a panel of sarcoma cell lines. Cell viabilities of 11 ES, 8 OS, and 3 RMS cell lines were measured after 72 hours of drug treatment. Cell viabilities of drug treated cells were normalized to those of cells grown in media without drug and fit to a dose-response curve to calculate IC50 values (Fig 4A). All cell lines were sensitive to buparlisib, with IC50 values that ranged from approximately 560 nM to 1.9 μM (S1 Table). In general, ES and RMS cell lines displayed IC50 values near 1.0 μM, while OS cell lines displayed slightly higher values (Fig 4B and S1 Table). MCF-7 cells containing the E545K *PIK3CA* mutation displayed a lower IC50 of 173 nM, despite having a similar phospho-Akt IC50 (S1B–S1D Fig, S1 Table). Additionally, IC50 values for buparlisib were higher than for the pan-PI3K inhibitor GDC-0941 in sarcoma cell lines (S3 Fig, S1 Table). Sensitivity to buparlisib treatment did not correlate with PTEN expression or basal phospho-Akt levels (Fig 1A and S1 Table). While the cell line with the lowest phospho-Akt IC50 was the most sensitive to buparlisib (A204), overall there was minimal correlation between Akt phosphorylation and cell viability IC50 values (Fig 4C). This agrees with a prior study that demonstrated increased buparlisib sensitivity only in cell lines with an activating *PIK3CA* mutation [21].

**Fig 5:**
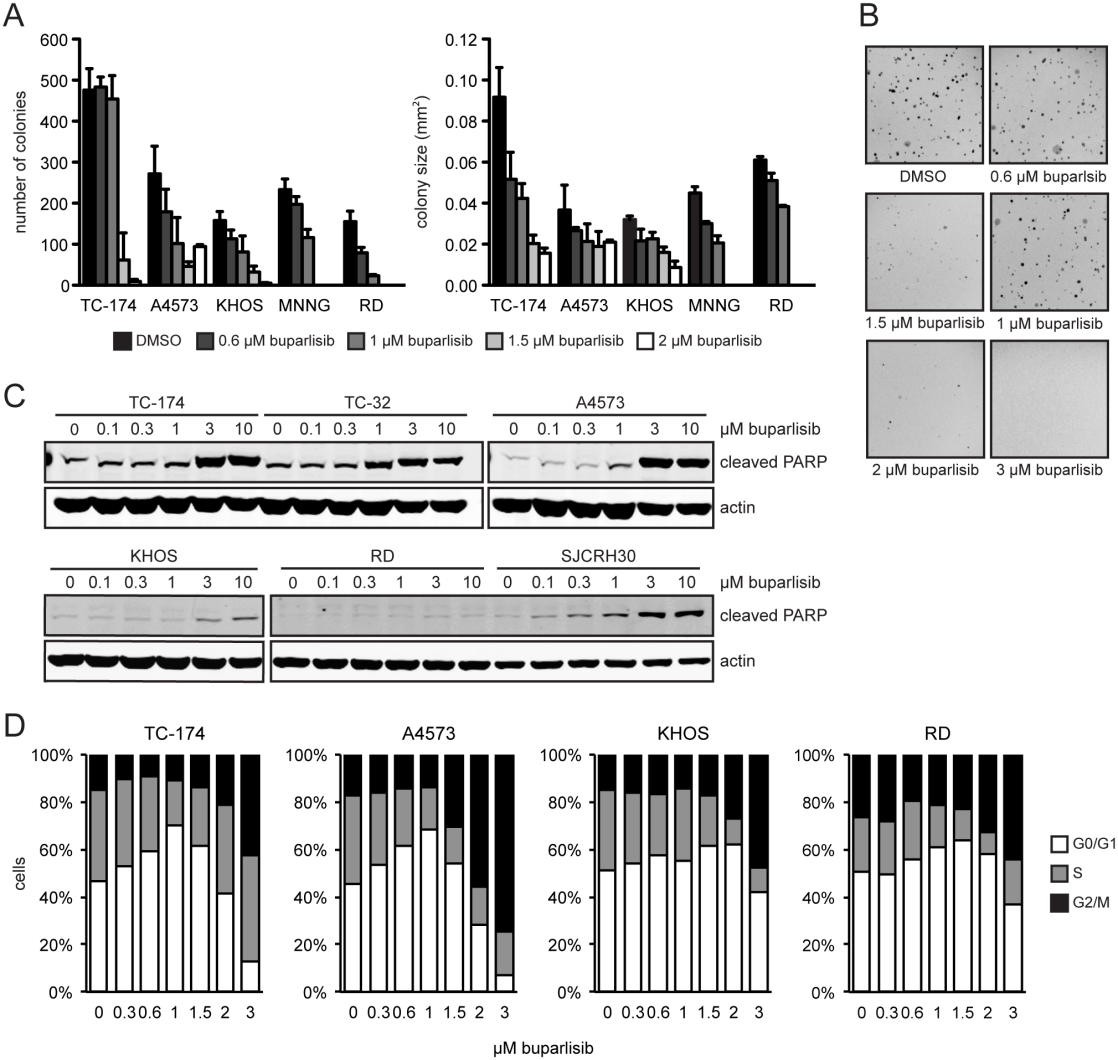
Buparlisib inhibits anchorage independent growth and induces apoptosis. (**A**) Plot of number and size of ES (TC-174, A4573), OS (KHOS, MNNG), and RMS (RD) colonies formed in soft agar in the presence of DMSO or increasing concentrations of buparlisib. Columns represent the average of three replicates, error bars represent standard deviation. (**B**) Representative pictures of TC-174 soft agar colonies. (**C**) Immunoblot analysis of cleaved PARP in ES (TC-174, TC-32, A4573), OS (KHOS), and RMS (RD, SJCRH30) treated with increasing doses of buparlisib for 24 hours. (**D**) Cell cycle analysis after 24 hours of buparlisib treatment of ES (TC-174, A4573), OS (KHOS), and RMS (RD) cell lines. Data are the mean of two independent experiments.

Buparlisib also inhibited cellular proliferation in anchorage-independent growth conditions. Increasing concentrations reduced both soft agar colony number and size (Fig 5A). Cell growth was inhibited within a narrower range than observed for anchorage dependent growth. Minimal effects were observed at concentrations less than 1 μM and no soft agar colony formation occurred at concentrations greater than 2 μM (Fig 5A and 5B).

To determine if buparlisib induces apoptosis in addition to inhibiting cellular proliferation, we utilized immunoblot analysis to monitor poly ADP ribose polymerase (PARP) cleavage. 24 hours of drug treatment resulted in increased PARP cleavage at concentrations greater than 1.0 μM (Fig 5C). While we observed potent induction of apoptosis in ES cell lines, the effects were modest in OS and RMS cell lines. Additionally, cell cycle analysis demonstrated that buparlisib treatment at concentrations greater than 1.0 μM resulted in an increase in the G2/M population (Fig 5D). This occurs as a result of the ability of buparlisib to inhibit tubulin polymerization at higher concentrations [26].

### Buparlisib synergizes with other molecularly targeted agents

While buparlisib demonstrated single agent activity across a panel of pediatric sarcoma cell lines, IC50 values were generally in the micromolar range. Additionally, observed reactivation of Akt within 24 hours of treatment and activation of compensatory signaling pathways suggested buparlisib might be more effective when used in combination with other agents.

**Fig 6:**
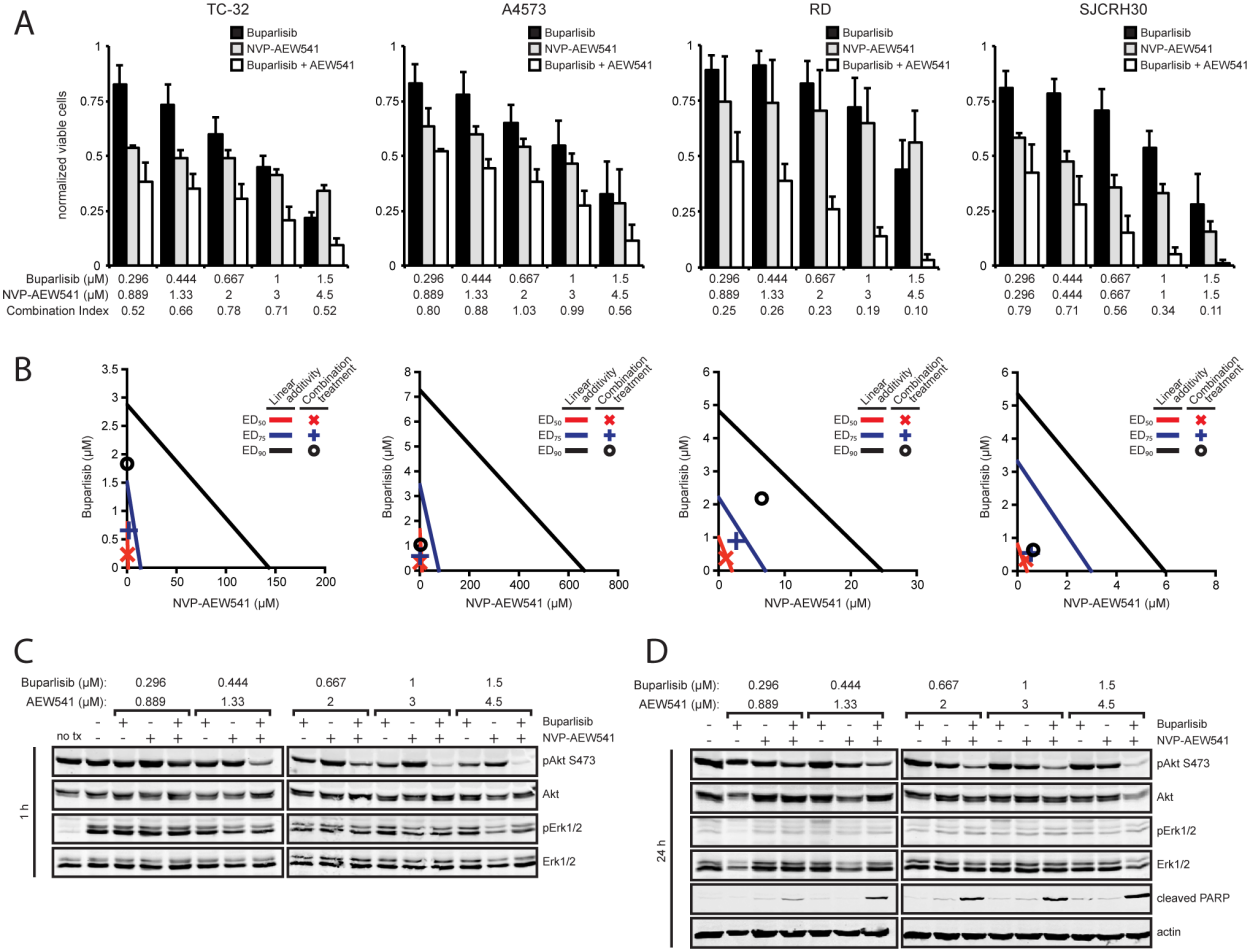
Buparlisib synergizes with NVP-AEW541 to further reduce Akt phosphorylation and augment apoptosis. (**A**) Cells were exposed to a series of 1.5-fold dilutions of buparlisib and NVP-AEW541 alone or in combination at a constant ratio of 1:3 (TC-32, A4573, RD) or 1:1 (SJCRH30) for 72 hours, then cell viability was determined by MTT assay. Columns represent the average of three independent experiments, error bars represent standard deviation. Combination index values greater than 1, equal to 1, or less than one indicate antagonism, additivity, or synergy. (**B**) Isobologram plot of the effect of buparlisib combined with NVP-AEW541. The effective doses of NVP-AEW541 and buparlisib are plotted on the x- and y-axis with lines of linear additivity connecting the ED_50_, ED_75_, and E_90_ for individual treatments. Points for combination treatment above, on, or below the lines indicate antagonism, additivity, or synergy, respectively. (**C**) Immunoblot analysis of phospho-Akt (S473), total Akt, phospho-Erk1/2 (T202/204 on Erk1, T185/187 on Erk2), and total Erk1/2 in A4573 cells after 1 hour of treatment with buparlisib, NVP-AEW541, or both. (**D**) Immunoblot analysis of phospho-Akt (S473), total Akt, phospho-Erk1/2 (T202/204 on Erk1, T185/187 on Erk2), total Erk1/2, cleaved PARP, and actin in A4573 cells after 24 hours of treatment with buparlisib, NVP-AEW541, or both.

Buparlisib was combined with chemotherapeutic and targeted agents to test for synergy. Sequential treatment of doxorubicin, a chemotherapeutic agent commonly used for the treatment of pediatric sarcomas, and buparlisib displayed additive results in ES and OS cell lines (S4 Fig). We also evaluated buparlisib in combination with the IGF1R inhibitor NVP-AEW541 based on the involvement of Akt signaling in resistance to anti-IGF1R therapy. Synergy was observed in ES and RMS cell lines (Fig 6A and 6B), while OS cell lines displayed additive effects (S5 Fig). The greatest synergistic effects were observed in the ES cell line A4573 and the RMS cell line SJCRH30 (Fig 6A and 6B), which display null or low PTEN expression (Fig 1A). We also measured the effects of combining buparlisib and NVP-AEW541 on Akt phosphorylation and induction of apoptosis (Fig 6C and 6D). The drug combination resulted in reduced phospho-Akt levels after one hour of treatment compared to each drug alone (Fig 6C). Furthermore, this response persisted up to 24 hours, indicating IGF1R inhibition can prevent Akt reactivation that was observed with buparlisib treatment alone (Fig 6D). Additionally, the drug combination resulted in increased apoptosis as measured by PARP cleavage at all dose levels after 24 hours of treatment (Fig 6D).

**Fig 7:**
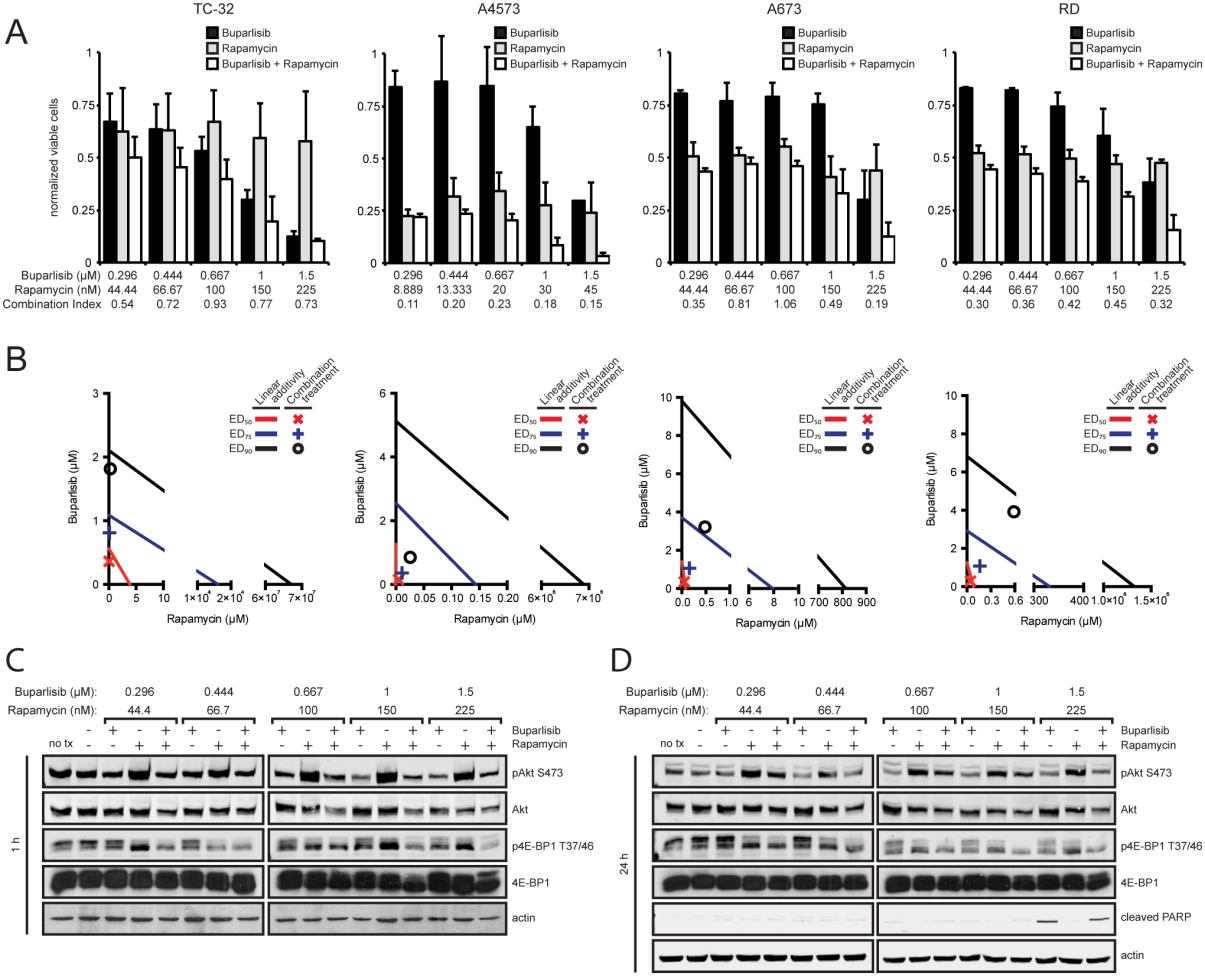
Buparlisib demonstrates increased synergy with rapamycin in PTEN deficient cell lines. (**A**) PTEN wild type (TC-32, A673, RD) or PTEN null (A4573) cell lines were exposed to a series of 1.5-fold dilutions of buparlisib and rapamycin alone or in combination at a constant ratio of 20:3 (TC-32, A673, RD) or 100:3 (A4573) for 72 hours, then cell viability was determined by MTT assay. Columns represent the average of three independent experiments, error bars represent standard deviation. Combination index values greater than 1, equal to 1, or less than one indicate antagonism, additivity, or synergy. (**B**) Isobologram plot of the effect of buparlisib combined with rapamycin. The effective doses of rapamycin and buparlisib are plotted on the x- and y-axis with lines of linear additivity connecting the ED_50_, ED_75_, and E_90_ for individual treatments. Points for combination treatment above, on, or below the lines indicate antagonism, additivity, or synergy, respectively. (**C**) Immunoblot analysis of phospho-Akt (S473), total Akt, phospho-4E-BP1 (T37/46), total 4E-BP1, and actin in A4573 cells after 1 hour of treatment with buparlisib, rapamycin, or both. (**D**) Immunoblot analysis of phospho-Akt (S473), total Akt, phospho-4E-BP1 (T37/46), total 4E-BP1, cleaved PARP, and actin in A4573 cells after 24 hours of treatment with buparlisib, rapamycin, or both.

While PTEN status did not correlate with sensitivity to buparlisib as a single agent, it appears lower PTEN expression may render cells more sensitive to buparlisib when combined with other agents that inhibit Akt signaling. Based on these results, we investigated whether combination with other targeted agents can display similar effects. Since Akt is also activated upon rapamycin treatment, we evaluated the effects of combining rapamycin with buparlisib. Synergy was observed in the four cell lines tested, with the greatest effects observed in A4573 cells (Fig 7A and 7B). The addition of buparlisib reduced rapamycin-mediated Akt activation and treatment with both drugs more potently suppressed phosphorylation of 4E-BP1 (Fig 7C and 7D). Unlike NVP-AEW541, the drug combination did not increase apoptosis (Fig 7D).

**Fig 8:**
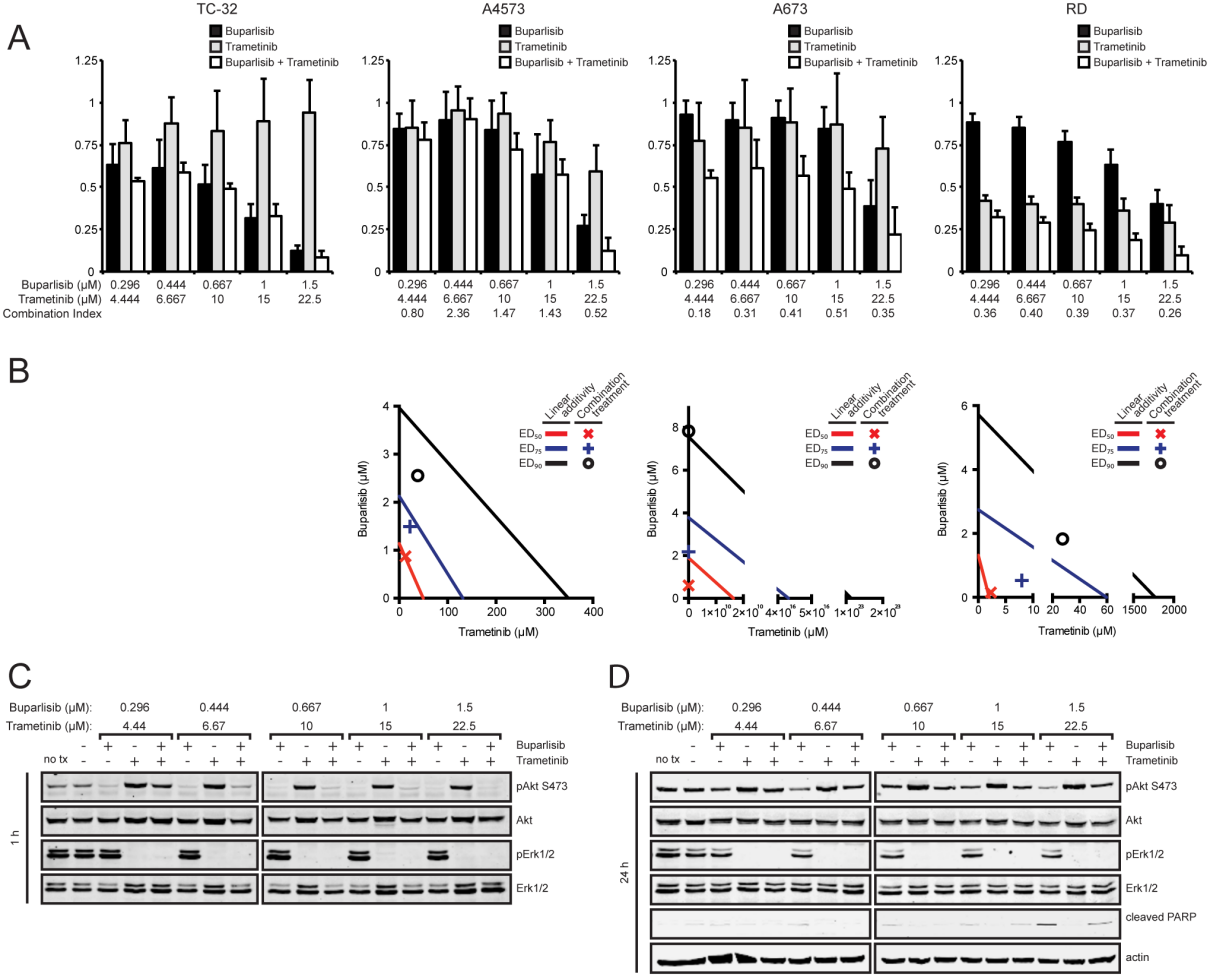
Buparlisib synergizes with trametinib in cell lines harboring MAPK pathway mutations. (**A**) Cell lines with (A673, RD) and without (TC-32, A4573) MAPK pathway mutations were exposed to a series of 1.5-fold dilutions of buparlisib and trametinib alone or in combination at a constant ratio of 1:15 for 72 hours, then cell viability was determined by MTT assay. Columns represent the average of three independent experiments, error bars represent standard deviation. Combination index values greater than 1, equal to 1, or less than one indicate antagonism, additivity, or synergy. (**B**) Isobologram plot of the effect of buparlisib combined with trametinib. The effective doses of trametinib and buparlisib are plotted on the x- and y-axis, respectively. Points for combination treatment above, on, or below the lines indicate antagonism, additivity, or synergy with lines of linear additivity connecting the ED_50_, ED_75_, and E_90_ for individual treatments. Isobologram could not be generated for TC-32 cells since no tramenitib cytotoxicity was observed. (**C**) Immunoblot analysis of phospho-Akt (S473), total Akt, phospho-Erk1/2 (T202/204, T185/187), and total Erk1/2 in A673 cells after 1 hour of treatment with buparlisib, trametinib, or both. (**D**) Immunoblot analysis of phospho-Akt (S473), total Akt, phospho-Erk1/2 (T202/204 on Erk1, T185/187 on Erk2), total Erk1/2, cleaved PARP, and actin in A673 cells after 24 hours of treatment with buparlisib, trametinib, or both.

MAPK mutations in sarcoma cell lines as well as increased Erk activity after buparlisib treatment suggested the addition of an agent targeting the MAPK pathway may also increase buparlisib efficacy. Buparlisib was combined with the MEK1/2 inhibitor trametinib in cell lines with (A673, RD) and without (TC-32, A4573) mutations in the MAPK pathway. Synergistic effects were only observed in cell lines that contained MAPK pathway mutations (Fig 8A and 8B). However, despite inhibition of both Akt and Erk phosphorylation when cells are treated with buparlisib and trametinib, no increase in apoptosis as measured by PARP cleavage was observed compared to treatment with buparlisib alone (Fig 8C and 8D).

## Discussion

Increased PI3K/Akt signaling has been demonstrated in pediatric sarcomas through modulation of IGF signaling and loss of PTEN expression. Additionally, the role of Akt in drug resistance suggests components of the PI3K/Akt pathway may provide novel therapeutic targets. In this study, we demonstrated that the pan-class I PI3K inhibitor buparlisib reduces cellular proliferation and induces apoptosis in various sarcoma cell lines. However, while initial buparlisib treatment reduced the activity of Akt and signaling molecules downstream of mTORC1, time course experiments revealed Akt reactivation within 24 hours. Drug combination experiments revealed synergy with a variety of molecularly targeted agents, indicating increased buparlisib efficacy when combined with other therapies. In addition to the observed synergy, combination with an IGF1R inhibitor prevented Akt reactivation and more potently induced apoptosis.

Reactivation of Akt within 24 hours of buparlisib treatment suggests possible removal of negative feedback inhibition. Transient inhibition of Akt phosphorylation was also observed with dual PI3K/mTOR inhibitor NVP-BEZ235, which was thought to be a result of loss of negative feedback through IRS1 [27]. While further analysis is required to fully elucidate this mechanism, the increase in phosphorylation at both phosphosites on Akt but not on S6K supports the involvement of a pathway upstream from mTOR. Moreover, the addition of an IGF1R inhibitor suppressed Akt phosphorylation after 24 hours of buparlisib treatment. Since IGF1R functions upstream of IRS1, inhibition at this point of the pathway can prevent IRS1 activation that can occur through release of negative feedback. We also observed we could increase the duration of time before Akt reactivation by increasing the amount of buparlisib. Additionally, we demonstrated that prolonged buparlisib treatment results in sustained down regulation of Akt phosphorylation, indicating that repeat dosing may also counteract Akt reactivation. However, concentrations of buparlisib used to prevent Akt reactivation were often greater than 1 μM. Since G2/M arrest occurs at these higher concentrations, this suggests PI3K-independent mechanisms are also contributing to cytotoxicity. These PI3K-independent effects also likely play a role in the increase in phospho-Erk levels that occurred with Akt reactivation since this response was also primarily observed at high buparlisib concentrations. Further investigation of the direct correlation between phospho-Akt levels over time and PI3K-dependent buparlisib cytotoxicity is warranted to fully define the role of Akt reactivation in resistance to buparlisib treatment.

We demonstrated that buparlisib inhibited cellular proliferation with a median IC50 value of 1.1 μM and apoptosis was induced at concentrations greater than 1.0 μM. All cell lines evaluated were sensitive to the drug regardless of oncogenic mutation status. However, distinct results were observed for specific mutations when buparlisib was combined with other agents. For example, cell lines with low PTEN expression displayed increased synergy when combined with an IGF1R inhibitor. Additionally, the PTEN null cell line A4573 demonstrated increased effects with the addition of rapamycin. Combination of buparlisib with trametinib only resulted in synergy in cell lines that harbored RAS or RAF mutations. These data suggest molecular profiling of tumors is important to determine which combination of agents will be most effective for an individual patient.

Other PI3K isoform specific and dual PI3K/mTOR inhibitors have been evaluated pre-clinically in pediatric sarcomas. The pan PI3K inhibitor SAR245408 demonstrated cytotoxic activity in a panel of pediatric tumor cell lines, including sarcomas, with a median relative IC50 of 10.9 μM [28]. IC50 values for ES and RMS cell lines ranged from 5.3 to 24.5 μM, which were five to 20-fold higher than the values we observed for buparlisib. The dual PI3K/mTOR inhibitor NVP-BEZ235 displayed more potent activity in pediatric sarcomas, with IC50 values ranging from approximately 6 to 500 nM [27]. Increased cytotoxicity compared to PI3K specific inhibitors was expected given that NVP-BEZ235 targets two components of the PI3K pathway. We observed similar results by combining buparlisib with rapamycin. While dual targeting and combination therapy both present therapeutic options, clinical evaluation of dual PI3K/mTOR inhibitors has revealed difficulties in optimizing the inhibition of distinct targets with a single pharmacokinetic profile. As a result, the use of two distinct agents may be more efficient due to the ability to optimize the dose of each drug separately. Additionally, two recent studies investigated the pre-clinical activity NVP-BEZ235 and NVP-BYL719, a p110α specific PI3K inhibitor, in osteosarcoma [29, 30]. Both agents inhibited *in vitro* cellular proliferation and tumor growth *in vivo*. Furthermore, combining NVP-BYL719 and the chemotherapeutic agent ifosfamide synergistically inhibited tumor growth. Overall, this promising pre-clinical data supports the rationale for clinical investigation of PI3K inhibitors in bone and soft tissue sarcomas.

While the cytotoxicity of buparlisib in a broad panel of sarcoma cell lines and its synergy with other targeted agents make it a good therapeutic candidate, PI3K-independent effects must also be taken into account. The initial characterization of the mechanism of action of buparlisib found that higher concentrations of the drug inhibit tubulin polymerization, which causes a block in progression from prometaphase to metaphase [26]. We also observed this effect in sarcoma cell lines, which displayed an increase percentage of cells in G2/M after 24 hours of buparlisib treatment at concentrations greater than 1 μM. Since some of our observed IC50 values are above 1 μM, cytotoxicity is likely due to a combination of PI3K inhibition and disruption of microtubule dynamics. Since cytotoxicity is observed with a discrete pan-PI3K inhibitor that does not inhibit tubulin polymerization, buparlisib cytotoxicity is likely not due to PI3K-independent effects alone. Though higher concentrations of buparlisib were required to elicit single agent activity, synergistic effects were observed at concentrations under 1 μM. Therefore, PI3K-independent effects can be minimized by utilizing combination therapy. Also, even as a single agent, doses of buparlisib required to elicit a clinical response *in vivo* did not increase mitotic index, suggesting in *vivo* tumor regression is solely due to PI3K inhibition [26].

Our results suggest that buparlisib will be most effective when combined with other targeted agents. Recent clinical testing in sarcoma has expanded to include drug combination studies. In a phase I study investigating the combination of the IGF1R antibody cixutmumuab and the mTOR inhibitor temsirolimus, an ES patient that developed resistance to a different IGF1R inhibitor achieved a complete response with combination therapy [31]. When this combination was evaluated in a phase II trial for patients with bone and soft-tissue sarcomas, a response rate of 15% was observed, including nine partial responses [32]. Buparlisib is also being evaluated in combination with other agents or as a second line therapy for drug resistant tumors. Current trials are investigating the ability to inhibit growth of tumors that have become resistant to mTOR inhibition as well as enhance response to tyrosine kinase inhibitors such as erlotinib (ClinicalTrials.gov; NCT01633060, NCT01487265).

The utility of buparlisib as an effective therapeutic agent is also influenced by the prevalence of activating *PIK3CA* mutations in sarcoma since increased single agent sensitivity is observed for tumors harboring these mutations [21]. Based on our data and recently published studies, PI3K mutations in sarcoma are rare. The PI3K status listed for the ES cell lines used in this study was obtained from a larger sequencing analysis of close to 40 ES cell lines, none of which were found to contain PI3K mutations. Additionally, Brohl et al. examined 55 ES tumors and 31 cell lines for PI3K mutations and only one cell line contained a *PIK3CA* G118D mutation [33]. Similar results were observed in OS, where only one *PI3KCA* mutation was found in a set of 59 OS tumors [34]. However, 24% of the tumors had mutations in the PI3K/mTOR pathway, including 5 *PTEN* deletions, suggesting the pathway rather than specific mutations plays a role in tumorigenesis. *PIK3CA* mutations have a higher rate in rhabdomyosarcoma, where a recent study identified a frequency of 5.4% in a sample of 147 tumors [35]. This further implies that buparlisib will be most useful in combination with other agents in for the treatment of pediatric sarcomas.

## Supporting Information

S1 Fig Buparlisib treatment of MCF-7 cells. (**A**) Immunoblot analysis of phospho-Akt (S473), total Akt, phospho-Erk1/2 (T202/T204 on Erk1, T185/T187 on Erk2), and total Erk in MCF-7 cells after treatment with 1 μM buparlisib for periods of 5 minutes to 24 hours. (**B**) MCF-7 cells were treated with media containing 0.1% DMSO or concentrations of buparlisib ranging from 10 nM to 10 μM for 72 hours. Cell viability was determined by MTT assay. Percent cell viability was plotted against log buparlisib concentration and IC50 values were calculated by fitting this data to a four-parameter, variable slope sigmoid dose-response model. Each point is the average of at three independent experiments. (**C**) Immunoblot analysis of phospho-Akt (S473), total Akt, phospho-Erk1/2 (T202/T204 on Erk1, T185/T187 on Erk2), and total Erk in MCF-7 cells treated with increasing concentrations of buparlisib for one hour. (**D**) Phospho-Akt (S473) and total Akt levels were quantitated based on Odyssey software integrated intensity values. Phospho-Akt levels were normalized to total Akt levels, then normalized to cells treated with DMSO in order to determine relative phospho-Akt levels. Relative phospho-Akt levels were plotted against log buparlisib concentration and IC50 values were calculated by fitting this data to a four-parameter, variable slope sigmoid dose-response model. Data points were derived from immunoblot in C.
(TIF)

S2 Fig Phospho-p70 S6K levels do not increase over time after buparlisib treatment. Immunoblot analysis of phospho-p70 S6K (T389) and total p70 S6K in ES (TC-174, A4573), OS (KHOS), and RMS (RD, A204) cells after treatment with buparlisib for periods of 5 minutes to 24 hours. A4573 cells were treated with 3 μM buparlisib. All other cell lines were treated with 1 μM buparlisib.
(TIF)

S3 Fig GDC-0941 inhibits proliferation of pediatric sarcoma cell lines. (**A**) Cells were treated with media containing 0.1% DMSO or concentrations of GDC-0941 ranging from 10 nM to 10 μM for 72 hours. Cell viability was determined by MTT assay. Percent cell viability was plotted against log GDC-0941 concentration and IC50 values were calculated by fitting this data to a four-parameter, variable slope sigmoid dose-response model. Each point is the average of at least two independent experiments. (**B**) Cell viability IC50 values for pediatric sarcoma cell lines. Columns represent the average of at least two independent experiments.
(TIF)

S4 Fig Combining buparlisib with doxorubicin displays additive effects. (**A**) ES (TC-32, A4573) and OS (KHOS) cells were exposed to a series of 1.5-fold dilutions of buparlisib (72h) and doxorubicin (4h) alone or in combination at a constant ratio of 20:3. For combination treatment, cells were treated with doxorubicin for 4 hours, then buparlisib for 72 hours. Cell viability was determined by MTT assay after 72 hours. Columns represent the average of at least two independent experiments, error bars represent standard deviation. Combination index values greater than 1, equal to 1, or less than one indicate antagonism, additivity, or synergy. (**B**) Isobologram plot of the effect of buparlisib combined with doxorubicin. The effective doses (ED) of doxorubicin and buparlisib are plotted on the x- and y-axis with lines of linear additivity connecting the ED_50_, ED_75_, and E_90_ for individual treatments. Points for combination treatment above, on, or below the lines indicate antagonism, additivity, or synergy, respectively.
(TIF)

S5 Fig Combining buparlisib with NVP-AEW541 displays additive effects in OS cells. (**A**) MNNG cells were exposed to a series of 1.5-fold dilutions of buparlisib and NVP-AEW541 alone or in combination at a constant ratio of 1:3 for 72 hours, then cell viability was determined by MTT assay. Columns represent the average of three independent experiments, error bars represent standard deviation. Combination index values greater than 1, equal to 1, or less than one indicate antagonism, additivity, or synergy. (**B**) Isobologram plot of the effect of buparlisib combined with NVP-AEW541. The effective doses of NVP-AEW541 and buparlisib are plotted on the x- and y-axis with lines of linear additivity connecting the ED_50_, ED_75_, and E_90_ for individual treatments. Points for combination treatment above, on, or below the lines indicate antagonism, additivity, or synergy, respectively.
(TIF)

S1 Table Buparlisib cell viability, Buparlisib phospho-Akt IC50 values, and GDC-0941 cell viability. (XLSX)
