# Supplementary figures and images for: Evaluation of In Vitro Activity of the Class I PI3K Inhibitor Buparlisib (BKM120) in Pediatric Bone and Soft Tissue Sarcomas
Source: PLoS One. 2015 Sep 24;10(9):e0133610. doi: 10.1371/journal.pone.0133610 (PMC4581723; doi:10.1371/journal.pone.0133610)

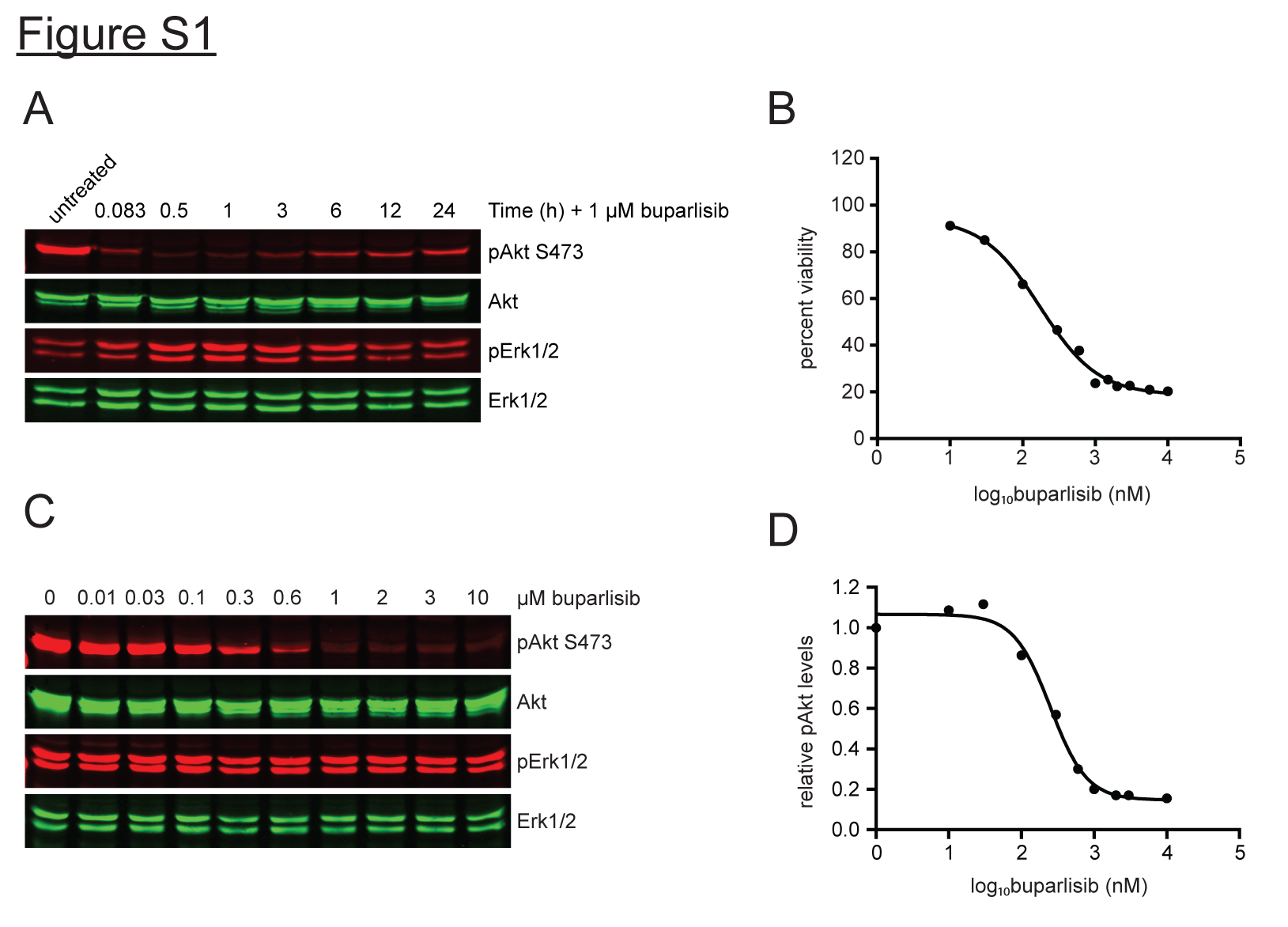

Supplement: S1 Fig — (A) Immunoblot analysis of phospho-Akt (S473), total Akt, phospho-Erk1/2 (T202/T204 on Erk1, T185/T187 on Erk2), and total Erk in MCF-7 cells after treatment with 1 μM buparlisib for periods of 5 minutes to 24 hours. (B) MCF-7 cells were treated with media containing 0.1% DMSO or concentrations of buparlisib ranging from 10 nM to 10 μM for 72 hours. Cell viability was determined by MTT assay. Percent cell viability was plotted against log buparlisib concentration and IC50 values were calculated by fitting this data to a four-parameter, variable slope sigmoid dose-response model. Each point is the average of at three independent experiments. (C) Immunoblot analysis of phospho-Akt (S473), total Akt, phospho-Erk1/2 (T202/T204 on Erk1, T185/T187 on Erk2), and total Erk in MCF-7 cells treated with increasing concentrations of buparlisib for one hour. (D) Phospho-Akt (S473) and total Akt levels were quantitated based on Odyssey software integrated intensity values. Phospho-Akt levels were normalized to total Akt levels, then normalized to cells treated with DMSO in order to determine relative phospho-Akt levels. Relative phospho-Akt levels were plotted against log buparlisib concentration and IC50 values were calculated by fitting this data to a four-parameter, variable slope sigmoid dose-response model. Data points were derived from immunoblot in C. (TIF) [file pone.0133610.s001.tif]

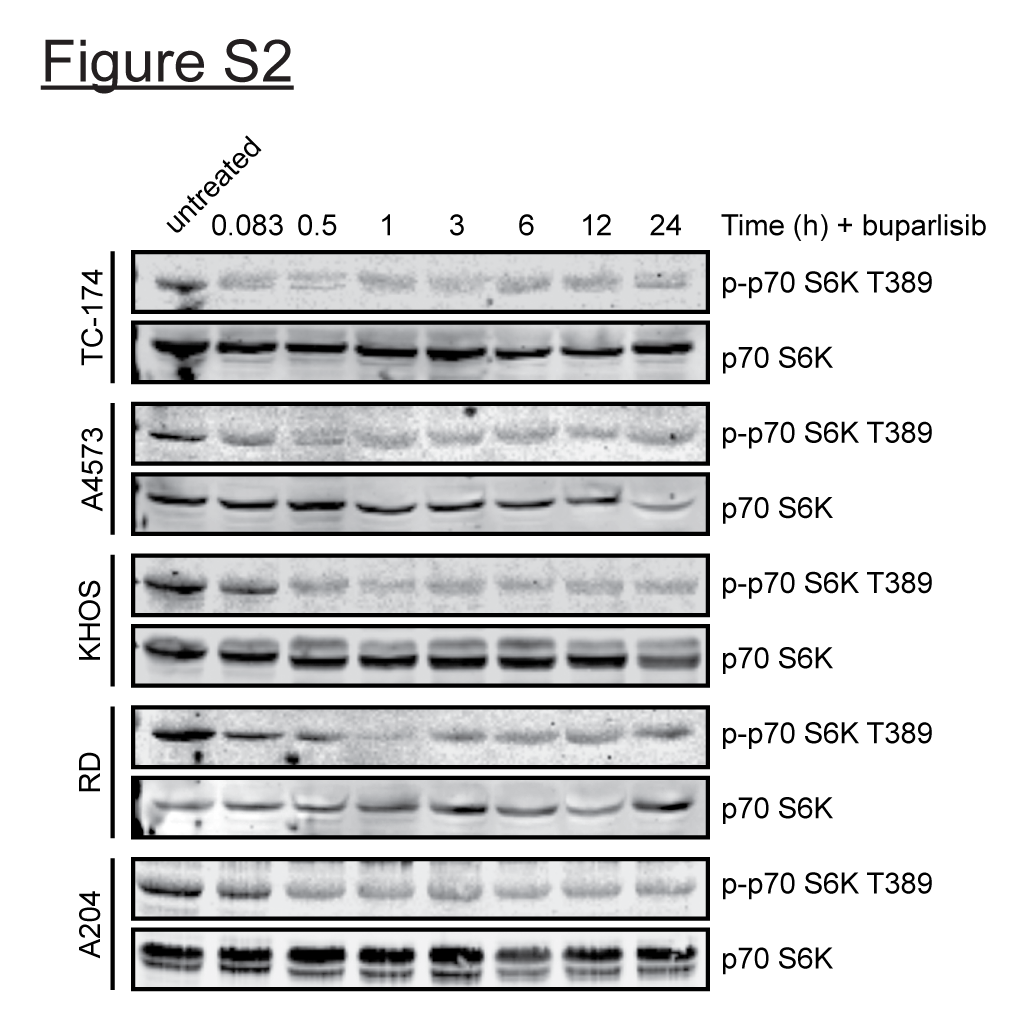

Supplement: S2 Fig — Immunoblot analysis of phospho-p70 S6K (T389) and total p70 S6K in ES (TC-174, A4573), OS (KHOS), and RMS (RD, A204) cells after treatment with buparlisib for periods of 5 minutes to 24 hours. A4573 cells were treated with 3 μM buparlisib. All other cell lines were treated with 1 μM buparlisib. (TIF) [file pone.0133610.s002.tif]

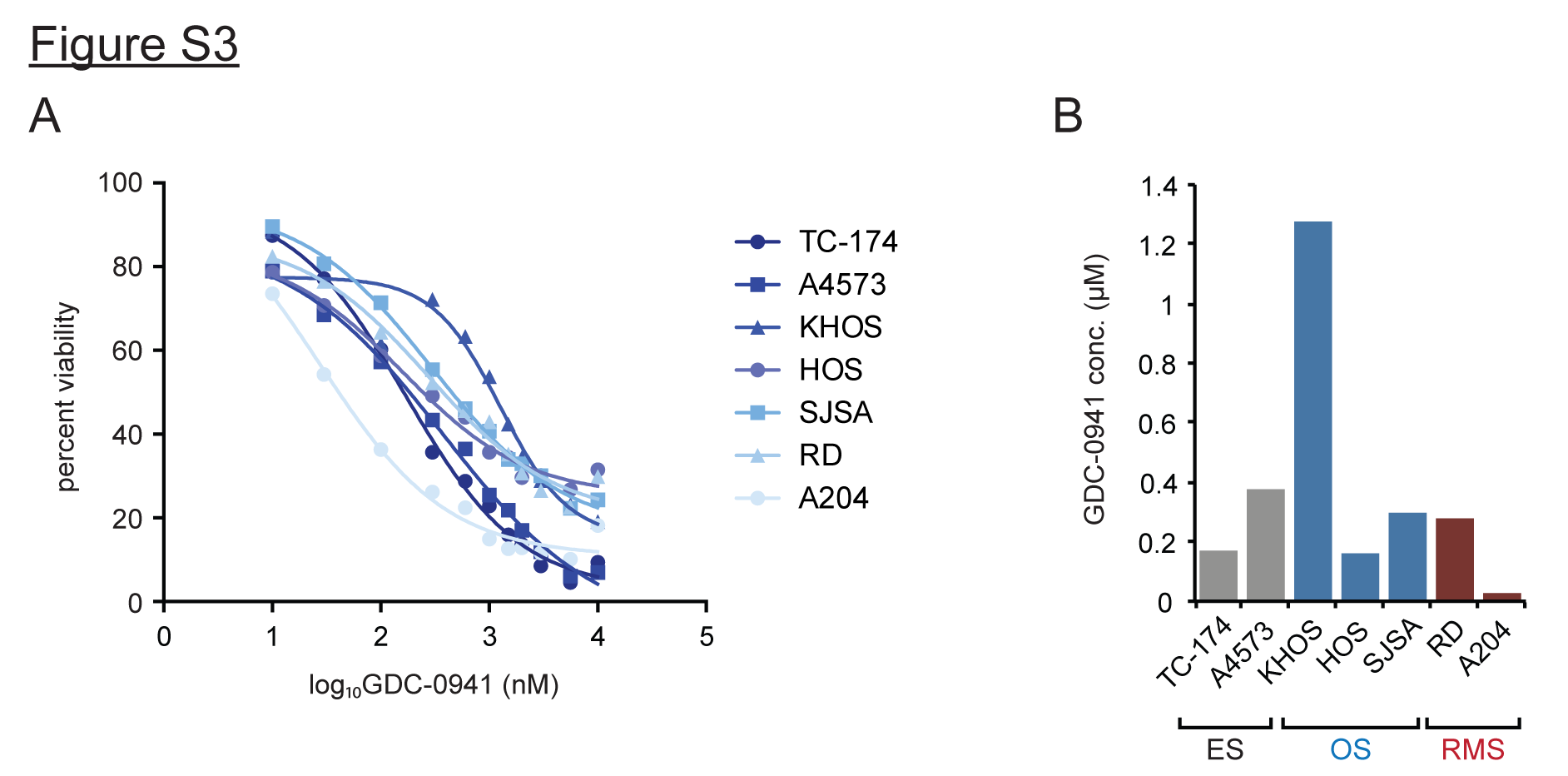

Supplement: S3 Fig — (A) Cells were treated with media containing 0.1% DMSO or concentrations of GDC-0941 ranging from 10 nM to 10 μM for 72 hours. Cell viability was determined by MTT assay. Percent cell viability was plotted against log GDC-0941 concentration and IC50 values were calculated by fitting this data to a four-parameter, variable slope sigmoid dose-response model. Each point is the average of at least two independent experiments. (B) Cell viability IC50 values for pediatric sarcoma cell lines. Columns represent the average of at least two independent experiments. (TIF) [file pone.0133610.s003.tif]

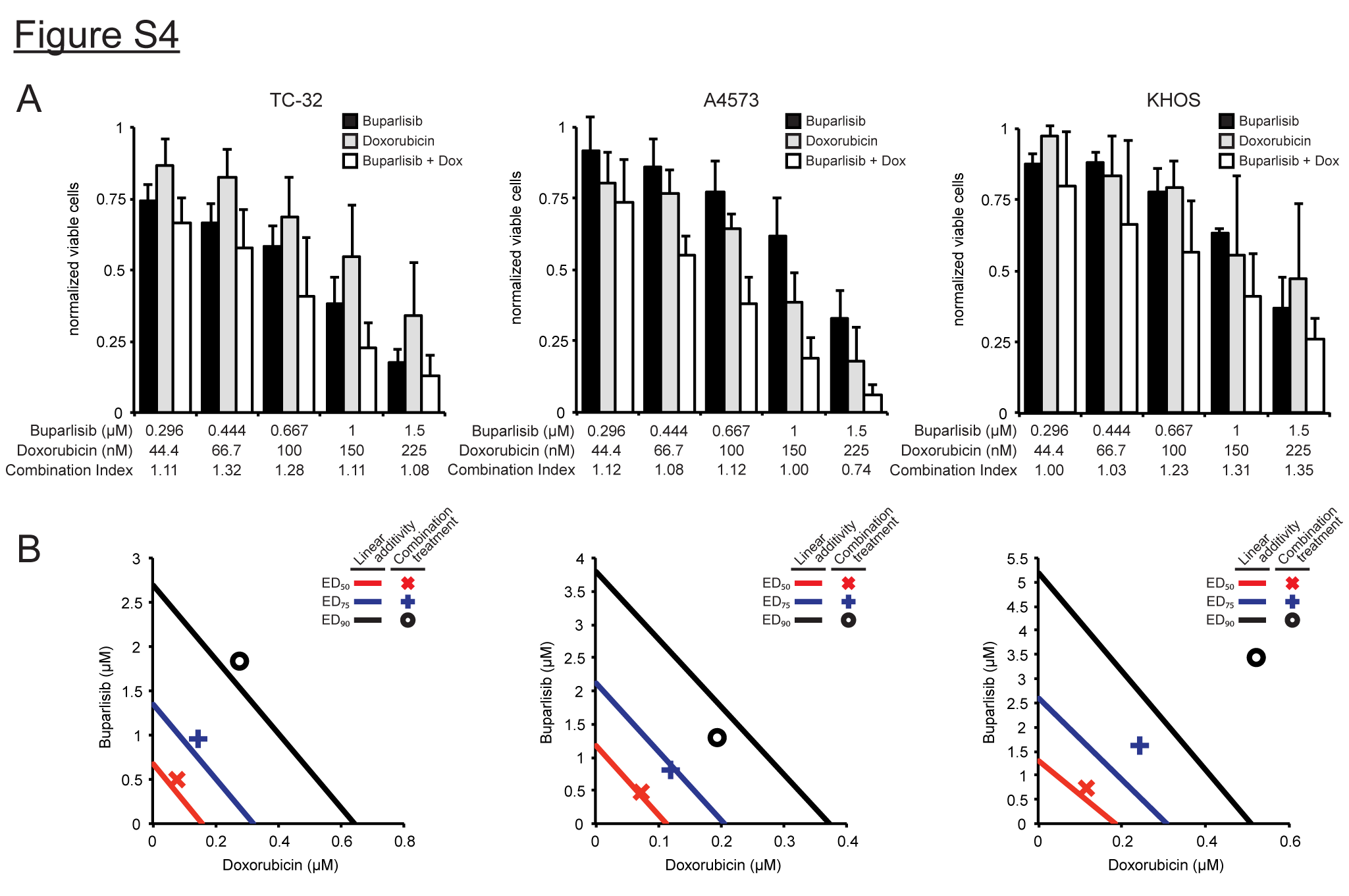

Supplement: S4 Fig — (A) ES (TC-32, A4573) and OS (KHOS) cells were exposed to a series of 1.5-fold dilutions of buparlisib (72h) and doxorubicin (4h) alone or in combination at a constant ratio of 20:3. For combination treatment, cells were treated with doxorubicin for 4 hours, then buparlisib for 72 hours. Cell viability was determined by MTT assay after 72 hours. Columns represent the average of at least two independent experiments, error bars represent standard deviation. Combination index values greater than 1, equal to 1, or less than one indicate antagonism, additivity, or synergy. (B) Isobologram plot of the effect of buparlisib combined with doxorubicin. The effective doses (ED) of doxorubicin and buparlisib are plotted on the x- and y-axis with lines of linear additivity connecting the ED50, ED75, and E90 for individual treatments. Points for combination treatment above, on, or below the lines indicate antagonism, additivity, or synergy, respectively. (TIF) [file pone.0133610.s004.tif]

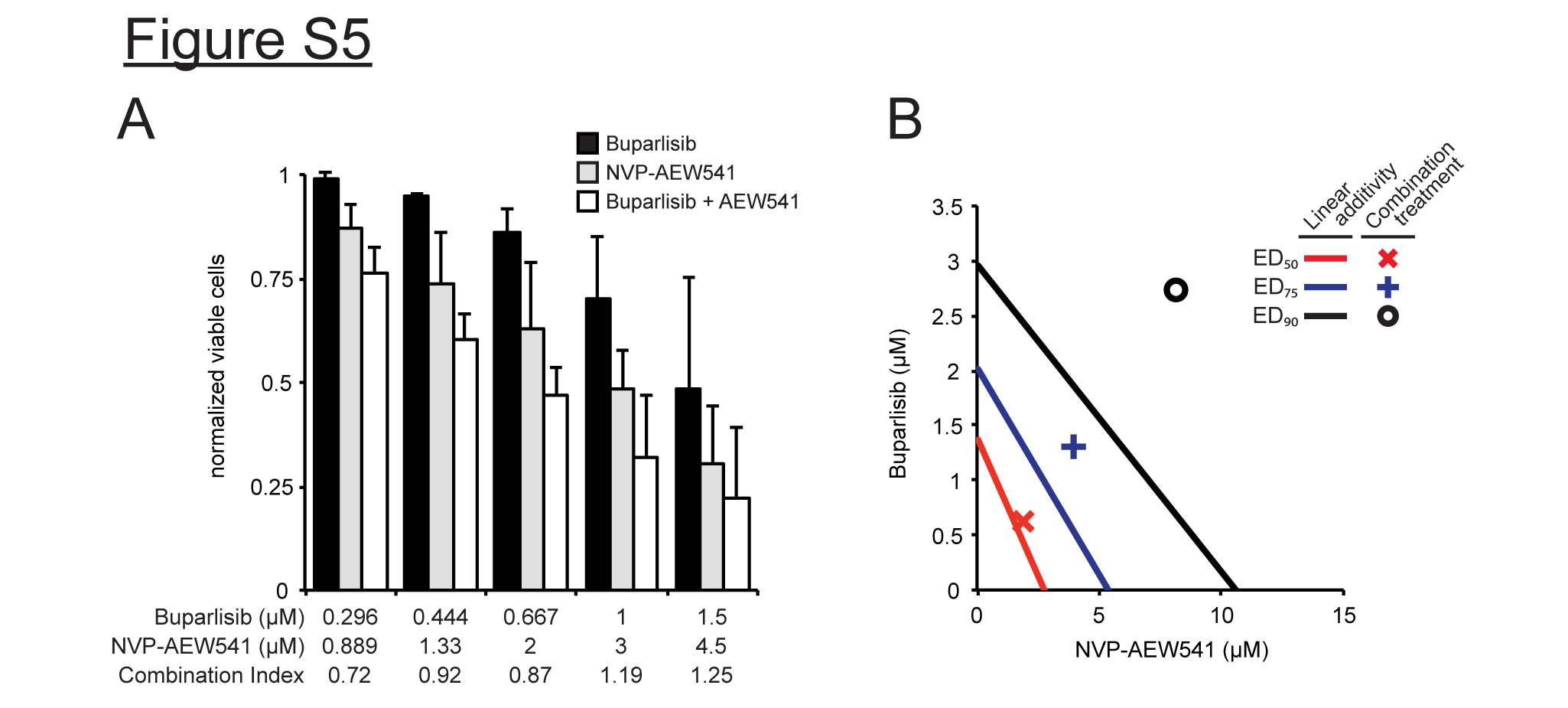

Supplement: S5 Fig — (A) MNNG cells were exposed to a series of 1.5-fold dilutions of buparlisib and NVP-AEW541 alone or in combination at a constant ratio of 1:3 for 72 hours, then cell viability was determined by MTT assay. Columns represent the average of three independent experiments, error bars represent standard deviation. Combination index values greater than 1, equal to 1, or less than one indicate antagonism, additivity, or synergy. (B) Isobologram plot of the effect of buparlisib combined with NVP-AEW541. The effective doses of NVP-AEW541 and buparlisib are plotted on the x- and y-axis with lines of linear additivity connecting the ED50, ED75, and E90 for individual treatments. Points for combination treatment above, on, or below the lines indicate antagonism, additivity, or synergy, respectively. (TIF) [file pone.0133610.s005.tif]
